# Construction of a prognostic risk model based on pyroptosis-related genes and comprehensive analysis of key genes and tumor immune microenvironment for colon cancer

**DOI:** 10.1097/MD.0000000000039300

**Published:** 2024-09-06

**Authors:** Mengxi Liu, Jin Zhang, Yu Zhao, Xiaoyi Zhang

**Affiliations:** a College of Chemistry and Life Science, Beijing University of Technology, Beijing, China.

**Keywords:** colon adenocarcinoma (COAD), mechanism, prognosis, pyroptosis, tumor immune microenvironment (TIME)

## Abstract

Pyroptosis-related genes have great potential for prognosis, an accurate prognostic model based on pyroptosis genes has not been seen in Colorectal adenocarcinoma (COAD). Furthermore, understanding the mechanisms of gene expression characteristics and the Tumor Immune Microenvironment associated with the prognosis of COAD is still largely unknown. Constructing a prognostic model based on pyroptosis-related genes, and revealing prognosis-related mechanisms associated with the gene expression characteristics and tumor microenvironment. 59 pyroptosis-related genes were collected. The gene expression data and clinical data of COAD were downloaded from The Cancer Genome Atlas. External validation datasets were downloaded from the Gene Expression Omnibus database. 10 characteristic genes with prognostic values were obtained using univariate and LASSO Cox. 10-gene Riskscore prognostic model was constructed. Both gene set enrichment analysis and network propagation methods were used to find pathways and key genes leading to different prognostic risks. The area under the ROC curves were used to evaluate the performance of the model to distinguish between high-risk and low-risk patients, the results were 0.718, 0.672, and 0.669 for 1-, 3-, and 5-year survival times. A nomogram based on Riskscore and clinical characteristics showed the probability of survival at 1, 3, and 5 years, and the calibration curves showed good agreement between the predicted and actual observations, its C-index is 0.793. The decision curves showed that the net benefit of the nomogram was significantly superior to that of the other single variables. Four key pathways leading to different prognostic risks were obtained. Six key genes with prognostic value, significant expression differences (*P* < .05) and significant survival differences (*P* < .05) between high/low risk groups were obtained from the gene set of all 4 key pathways. This study constructed a prognostic model for COAD using 10 pyroptosis-related genes with prognostic value. This study also revealed significant differences in specific pathways and the tumor immune microenvironment (TME) between the high-risk group and the low-risk group, highlighted the roles of ALDH5A1 and Wnt signaling in promoting COAD and the suppressive effects of the IL-4/IL-13 pathway and RORC on COAD. The study will be helpful for precision therapy.

## 1. Introduction

Colorectal cancer (CRC) was the third most commonly diagnosed cancer worldwide and the second most common cause of death in new cancer cases in 2020.^[1]^ CRC is divided into 2 types: colon adenocarcinoma (COAD) and rectum adenocarcinoma, of which approximately 98% of patients have COAD.^[2]^ Since the 2 types have distinct differences in genomic features and clinical management, this study only focused on COAD.

Early symptoms of COAD are not obvious, leading to quite a low diagnostic rate, most patients are already in advanced stages when they are first diagnosed with COAD. Patients in advanced stages have rapid disease progression and poor prognosis. The 5-year survival rate for stage I patients is as high as 91%, while only 14% for stage IV.^[3]^ Tumor is highly heterogeneity, even if patients are at the same stage with similar treatment strategies, their clinical outcomes may also vary widely due to heterogeneity.^[4]^ Therefore, if more appropriate and personalized clinical treatment strategies are used for patients in a timely determination of prognosis, the recurrence rate will be decreased and may even reach the standard of clinical healing. Developing accurate prognostic models are important for the treatment and improvement of the survival rate of COAD.

Pyroptosis is a form of programmed cell death.^[5]^ Compared with apoptosis, pyroptosis is more rapid and accompanied by the release of large amounts of pro-inflammatory factors.^[6]^ Many biological processes in tumor cells are accompanied by pyroptosis, pyroptosis promotes or suppresses tumor progression. For example, gasdermin E (*GSDME*)-mediated pyroptosis released high mobility group box 1 protein, which induced tumor cell proliferation through the ERK1/2 pathway, while Granzyme A in cytotoxic lymphocytes cleaved the lysine at positions 244 and 229 of the gasdermin B (*GSDMB*) protein in tumor cells, activated *GSDMB*-induced pyroptosis, thus killed tumor cells.^[7]^ As can be seen from the above studies, pyroptosis is a double-edged sword for tumors.

Pyroptosis shows an association with the activation or inhibition of numerous signaling pathways, therefore, pyroptosis-related genes have great potential for diagnosis, prognosis, and personalized targeted therapy. There have been several studies confirming the significance of pyroptosis in the prognosis and treatment of tumors. For example, pyroptosis-related gene gasdermin C（*GSDMC*）was found to be a prognostic factor in patients with lung adenocarcinoma or ovarian cancer.^[8,9]^ Other studies pointed out that pyroptosis-related genes can be used as therapeutic targets. *GSDME* is mainly highly expressed in normal cells and is low in tumor cells due to the hypermethylation of its promoter.^[10]^ Decitabine, a DNA methyltransferase Inhibitor, can inhibit hypermethylation in the *GSDME* promoter region, promote tumor cell pyroptosis, and enhance the immune effect, thus inhibiting tumor growth.^[11]^ These studies all confirmed the potential of pyroptosis-related genes in the prognostic stratification and as therapeutic targets. So far, there are relatively few studies focused on pyroptosis of COAD.^[12–20]^

In addition, pyroptosis-related outcomes were correlated with different immune microenvironments. The tumor microenvironment (TME) consists of tumor cells, immune cells, endothelial cells, and extracellular matrix, as well as their secreted cytokines.^[21]^ The metabolic status of immune cells in the TME is a key factor affecting their normal immune response, and increasing evidence suggest that the tumor immune microenvironment (TIME) plays an important role in CRC development and cancer immunotherapy. On the one hand, as an innate immune mechanism, it can inhibit tumor development, on the other hand, the inflammatory factors released during pyroptosis can form a microenvironment suitable for tumor growth.^[22,23]^ In a word, pyroptosis plays an important and complex role in tumor progression and prognosis, and the process is also affected by the immune microenvironment. Tumors and TIME are a closely linked and inseparable whole, and the strategic innovation of next-generation tumor precision medicine is expected to be achieved by reshaping TIME. In recent years, immunotherapy has become a research hotspot in the field of tumor therapy, and a variety of therapeutic strategies targeting TIME, including immune checkpoint inhibitors (ICIs), have achieved some success in CRC. Many other studies have also shown that the TIME is closely related to the prognosis of CRC. Chu et al computationally screened 14 immune checkpoint genes and validated them with IF, HE, and IHC and found that inducible T-cell stimulating factor (ICOS) was highly correlated with the survival and clinical characteristics of CRC patients.^[24]^ The increased presence of tumor-infiltrating dendritic cells (DCs) in head and neck cancer, NSCLC, and CRC has been associated with improved progression-free survival.^[25–27]^ These studies indicated that several immune cells are associated with prognostic of CRC. Understanding the infiltration levels and functions of different types of immune cells in the TIME of CRC is important for evaluating the prognosis of CRC patients treated with immunotherapy, exploring potential therapeutic targets, and guiding clinicians to provide more individualized treatments for CRC patients.^[28]^ Nowadays, the mechanisms, gene expression characteristics, and TIME associated with the prognosis of COAD are still largely unknown.

In this study, a gene set would be constructed by collecting pyroptosis-related genes from multiple databases and literature. And then, a reliable prognostic model for COAD based on these pyroptosis-related gene sets would be developed. Further, the key pathways and key genes, mechanisms, and TIME-related to the prognosis of COAD were analyzed. The study will provide insights into the treatment and drug development of COAD.

## 2. Method

### 2.1. Construction of a pyroptosis-related gene set

Genes involved in pyroptosis were collected from several literature^[29–36]^ and databases, including MSigDB 3.0 (https://www.gsea-msigdb.org/gsea/msigdb/index.jsp),^[37]^ GO (http://geneontology.org/),^[38]^ KEGG (https://www.kegg.jp/),^[39]^ and REACTOME (https://www.joaskin.com/).^[40]^ (up to December 2022).

### 2.2. Patient selection

Selected patients met the following selection criteria: i) The samples with no duplicates; ii) No lack of survival data; iii) Survival times longer than 30 days.

### 2.3. Data download and pre-processing

The gene expression data (FPKM) and clinical data of COAD were downloaded from The Cancer Genome Atlas (TCGA, https://portal.gdc.cancer.gov/, accessed in 2022). Statistcal analysis was performed using R language (version 4.0; www.r-project.org). The messenger RNA (mRNAs) with less than 20% missing data in all samples were removed. The outliers (|Z-score| > 3) identified using the Z-score method were replaced with the median. The expression profile data were normalized using the algorithm log(TPM + 1).

As an external validation set, the dataset GSE17536 with the platform GPL570 was downloaded from the Gene Expression Omnibus (GEO, https://www.ncbi.nlm.nih.gov/geo/) database.^[41]^ The datasets GSE41258 with the platform GPL96 and GSE40367 with the platform GPL570 were also downloaded from the GEO database to evaluate the ability of the prognosis model to predict the metastasis of COAD patients. The samples with missing clinical data in the above datasets were also removed.^[42,43]^

### 2.4. Construction and validation of a prognostic model based on pyroptosis-related genes

#### 2.4.1. Construction of a prognostic model

The univariate Cox regression analysis was used to select genes with prognostic value (*P* < .20) from the constructed pyroptosis gene set. The LASSO Cox regression analysis was performed to further reduce the dimensionality using the R package “glmnet,” which was able to eliminate false positives due to overfitting, and finally, the characteristic genes included in the optimal model were determined by ten-fold cross-validation. Based on the characteristic genes, the following equation was used to construct the prognostic model (formula 1):


$$\begin{array}{l} Riskscore=\sum_{i}^{n}X_{i}*Y_{i} \end{array}$$ (1)

In the equation, the “n” denoted the number of genes included in the model, the “X” denoted the LASSO Cox regression coefficient of the gene, and the “Y” denoted the expression data of the gene. The Riskscore was calculated for each sample in the TCGA dataset and the GEO external dataset based on the constructed prognostic model, and the samples were divided into the high-risk group and the low-risk group according to the median of all the Riskscores.

#### 2.4.2. Validation of a prognostic model

To validate the abilities of the prognostic model to differentiate high-risk samples from low-risk samples and predict the survival rate, the survival analysis using the R package “survminer” and the receiver operating characteristic (ROC) curves using the R packages “survival” and ”time ROC” were performed. The above analyses were performed using the TCGA dataset and GSE17536.

The GEO external datasets GSE41258 and GSE40367 were used to evaluate the performance of the prognostic model in predicting the metastasis of COAD. To solve the data-imbalance problem in the GSE41258, the primary COAD samples were randomly divided into several groups, each group had the same number of samples with liver and lung metastasis. Then, each group was divided into the train set and the test set according to the ratio of 7:3. At last, the ability of the prognostic model to differentiate primary COAD samples from samples with metastasis in each group was calculated by the random forest method, respectively. The mean diagnostic performance was obtained. In the GSE40367 dataset, the sample sizes of primary COAD and metastatic COAD were similar, the predictive ability of the prognostic model was calculated directly using random forest, and samples were divided into training and test sets according to 7:3.

To verify whether the Riskscore can be used as an independent predictor for clinical applications, univariate and multivariate Cox analyses were performed with the Riskscore and clinical characteristics (sex, age, and tumor stage) based on the TCGA dataset and the GSE17536 dataset. The Riskscore and clinical characteristics were used to construct a nomogram that could predict the 1-, 3-, and 5-year survival rates of patients using the R packages ”rms” and “survival.” The calibration curves were used to evaluate the predictive ability of the nomogram of the R packages “rms” and “survival.” The Decision Curve Analysis (DCA) was used to assess the clinical net benefit of the nomogram using the R package “DCA.”

### 2.5. Pathway enrichment and tumor microenvironment analysis of high-risk and low-risk groups

The cancer-related mechanisms and TME were still largely unknown. To explore the underlying reasons for the differences in prognosis between the high-risk and low-risk groups, the pathways and TME involved in each group were further explored.

#### 2.5.1. Pathway enrichment analysis

In order to determine the respective pathways for high-risk and low-risk groups, 2 kinds of pathway enrichment analyses were done.

##### 2.5.1.1. Pathway enrichment using the GSEA method

Enrichment analysis was performed using gene set enrichment analysis (GSEA 4.2.3) for both high-risk and low-risk groups to determine the respective pathways. To be specific, firstly, all genes were ranked by the GSEA algorithm according to the extent of differences between high-groups and low-risk groups in gene expression, the higher the difference, the higher the ranking level, so the important genes were obtained based on the ranking scores. Then, those that contributed the most to the Enrichment score were identified as core genes, also known as the leading edge subset. For gene sets with a positive Enrichment score, the core genes were those before the peak, for gene sets with a negative Enrichment score, the core genes were those after the peak. Lastly, the pathways where the core genes were enriched were obtained. The reference gene set was “c2.cp.v7.4.symbols,” and pathways with NOM *P* < .05, FDR q < .25, and |NES| > 1 were selected.

##### 2.5.1.2. Pathway enrichment using network propagation

The human protein-protein interaction (PPI) network was downloaded from the STRING (https://www.string-db.org/) database. The largest connected component composed of the pairs with interaction scores larger than 700 in the PPI was selected as the key sub-network. The genes included in the prognostic model were considered as the seeds with a weight of 1 and other genes with a weight of 0, and the parameter “damping factor” was set to 0.85. The influence score of each gene was calculated by the network propagation method using the R package “PageRank,” and the top 100 genes with the highest influence scores were selected as the proximal genes. Pathway enrichment was performed based on the prognostic model genes and the proximal genes using the hypergeometric distribution, where the reference gene set was also “c2.cp.v7.4.symbols” and *P* < .001 was considered significant.

##### 2.5.1.3. The final results of pathway enrichment

The intersection of pathways between the above 2 methods was chosen for high-risk and low-risk groups, respectively. All the contained in those pathways were core genes.

#### 2.5.2. Selection of the key genes and their pathways

For the purpose of identifying key genes and pathways among the numerous pathways obtained above, the following 2 steps were carried out.

First, genes with prognostic value would be selected from core genes. The genes with important effects on the outcome variable (death) were obtained using univariate and multivariate Cox analysis separately for high-risk and low-risk groups. Threshold is *P* < .05. Second, genes with significant expression differences (*P* < .05) and significant survival differences (*P* < .05) between the 2 groups were selected as key genes separately for high-risk and low-risk groups. Among those different genes, upregulated genes were selected.

The intersections of the above 2 steps were selected as the key genes for high-risk and low-risk groups separately. The pathways in which the key genes were involved were the key pathways.

#### 2.5.3. Analysis of the mechanisms of the key genes and the key pathways

The role of key genes in each pathway and their contribution to COAD (pro- or anti-COAD) were analyzed, and the consistencies in their expression and survival analysis were validated.

## 3. Results

### 3.1. Construction of pyroptosis-related gene set

A total of 61 pyroptosis-related genes were collected. Among them, *ELANE* and *NLRP7* were found with more than 20% missing data in all samples, therefore, the remaining 59 genes were used as pyroptosis-related gene set for the subsequent analysis in this study. Their symbols, official names, and sources were shown in Table S1, Supplemental Digital Content, http://links.lww.com/MD/N468.

### 3.2. Patient selection

**Figure 1.**
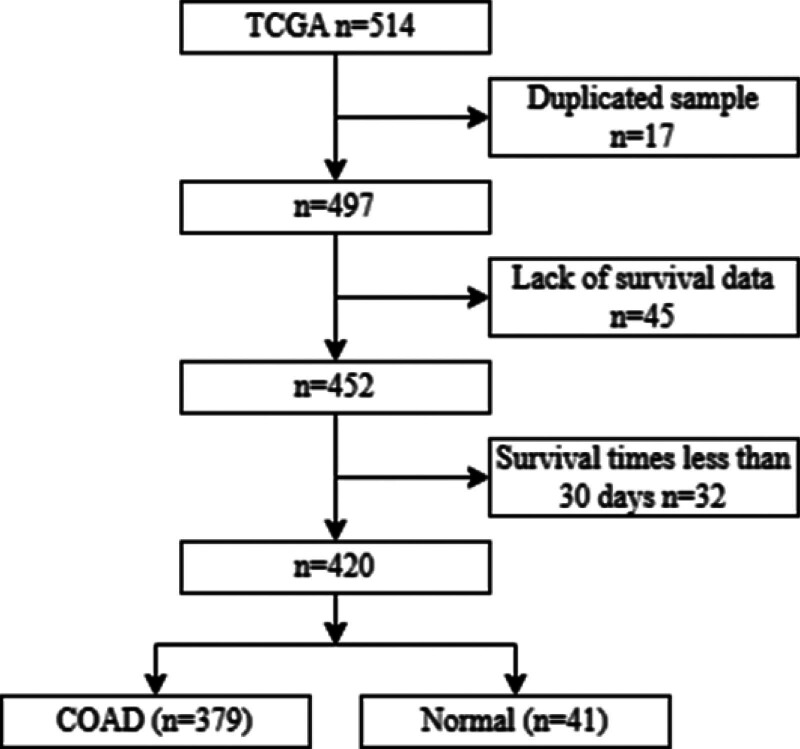
Flowchart depicting the patient selection process. COAD = colon adenocarcinoma, TCGA = The Cancer Genome Atlas.

In the TCGA dataset, the raw data contained a total of 514 samples. After pre-processing, a total of 379 COAD samples, 41 normal samples (Table 1, Figure 1).

**Table 1:** Clinical characteristics of the COAD patients and normal controls.

Parameters	COAD (n = 379)	Normal (n = 41)
Age (year)		
Range	31–89	40–90
Mean (SD)	66.3 (12.7)	70.3 (13.2)
Gender		
Male	202	20
Female	177	21
Stage		
Ⅰ	68	5
Ⅱ	147	22
Ⅲ	110	7
Ⅳ	54	7
Race		
White	179	17
Black or African American	52	3
Asian	11	1
Not reported	137	20

COAD = colon adenocarcinoma.

### 3.3. Data download and pre-processing

In the TCGA dataset, the raw data contained a total of 514 samples and 60483 mRNAs. After pre-processing, 15,841 genes containing 59 pyroptosis-related genes were obtained.

In the external validation dataset GSE17536, a total of 177 COAD samples and 20,484 genes were obtained. GSE41258 contained 186 primary COAD samples, 47 liver metastasis samples, and 20 lung metastasis samples. GSE40367 contained 13 primary COAD samples and 11 liver or lung metastasis samples.

### 3.4. Construction and validation of the prognostic model

#### 3.4.1. Construction of the prognostic model

The prognosis-related pyroptosis genes were obtained based on the expression data and clinical data (death) of 59 pyroptosis genes using univariate Cox analysis (*P* < .2) (Table S2, Supplemental Digital Content, http://links.lww.com/MD/N469).

**Figure 2.**
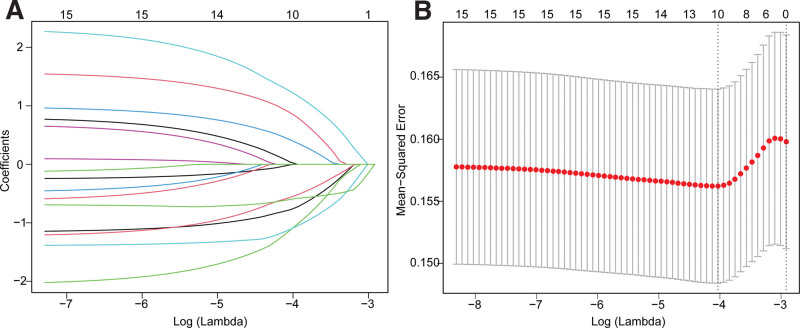
LASSO regression of 15 pyroptosis genes. (A) LASSO regression coefficient changes of 15 prognosis-related pyroptosis genes. (B) Tenfold cross-validation results for parameter selection in LASSO regression.

A total of 15 genes were selected for the following LASSO Cox regression to develop a robust prognostic model. The results showed that the penalty parameter (λ) was the smallest and the model was the most stable when 10 genes were included in the model (Fig. 2B). The 10 genes were: *CHMP7*, *CHMP6*, *CASP6*, *TP53*, *TNF*, *IRF1*, *PKN1*, *NOD1*, *CHMP4A*, and *GPX4*. Based on their respective regression coefficients (Fig. 2A), the prognostic model obtained was:

$$\begin{array}{l} \begin{array}{lllll} & Riskscore=\;\;\;-0.7107*CHMP7+1.4509\; & *PKN1-1.9004*CHMP6+1.0426*NOD1-0.6746\; & *CASP6-0.4343*TP53-0.6807*TNF-0.3589\; & *IRF1+0.9612*CHMP4A+0.8347*GPX4\; \end{array} \end{array}$$  (2)

Among them, the hazard ratios (HRs) of *CHMP7*, *CHMP6*, *CASP6*, *TP53*, *TNF*, and *IRF1* were less than 1, which were protective factors and associated with a better prognosis; HRs of *PKN1*, *NOD1*, *CHMP4A,* and *GPX4* were larger than 1, which were risk factors and associated with a poorer prognosis.

#### 3.4.2. Validation of the prognostic model

##### 3.4.2.1. Validation of the ability to differentiate high-risk samples from low-risk samples

**Figure 3.**
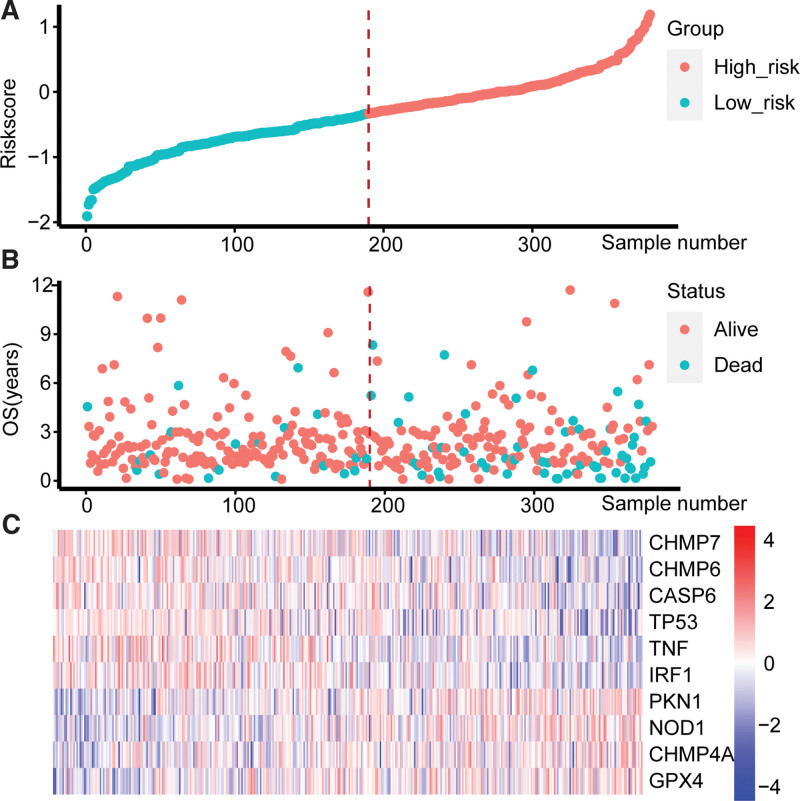
Riskscores, status, and gene expression levels of the high-risk group and low-risk group in the TCGA dataset. (A) Riskscore distribution of high-risk groups and low-risk. (B) Survival status of high-risk group and low-risk group. (C) Heatmap of the expression of genes within the model. CASP6 = Caspase 6, CHMP4A = Charged Multivesicular Body Protein 4A, CHMP6 = Charged Multivesicular Body Protein 6, CHMP7 = Charged Multivesicular Body Protein 7, GPX4 = Glutathione Peroxidase 4, IRF = Interferon Regulatory Factor, NOD1 = Nucleotide Binding Oligomerization Domain Containing 1, OS = Overall Survival, PKN1 = Protein Kinase N1, TNF = Tumor Necrosis Factor, TP53 = Tumor Protein P53.

**Figure 4.**
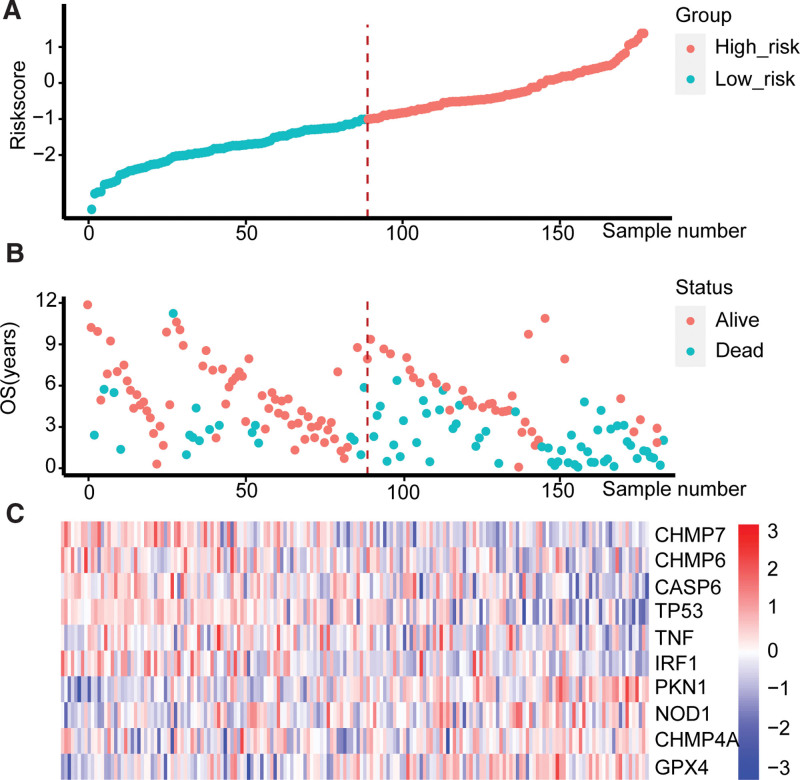
Riskscores, status, and gene expression levels of the high-risk group and low-risk group in the GSE17536 dataset. (A) Riskscore distribution of high-risk group and low-risk. (B) Survival status of high-risk group and low-risk group. (C) Heatmap of the expression of genes within the model. CASP6 = Caspase 6, CHMP4A = Charged Multivesicular Body Protein 4A, CHMP6 = Charged Multivesicular Body Protein 6, CHMP7 = Charged Multivesicular Body Protein 7, GPX4 = Glutathione Peroxidase 4, IRF = Interferon Regulatory Factor, NOD1 = Nucleotide Binding Oligomerization Domain Containing 1, OS = Overall Survival, PKN1 = Protein Kinase N1, TNF = Tumor Necrosis Factor, TP53 = Tumor Protein P53.

The Riskscore was calculated for each sample of the TCGA dataset and the external dataset GSE17536. Then all the samples were divided into the high-risk group and the low-risk group by median (median Riskscore is -0.3325129 for the TCGA dataset and -1.00518 for the GSE17536). The Riskscore, survival status, and gene expression for the 2 groups are shown in (Figs. 3 and 4).

**Figure 5.**
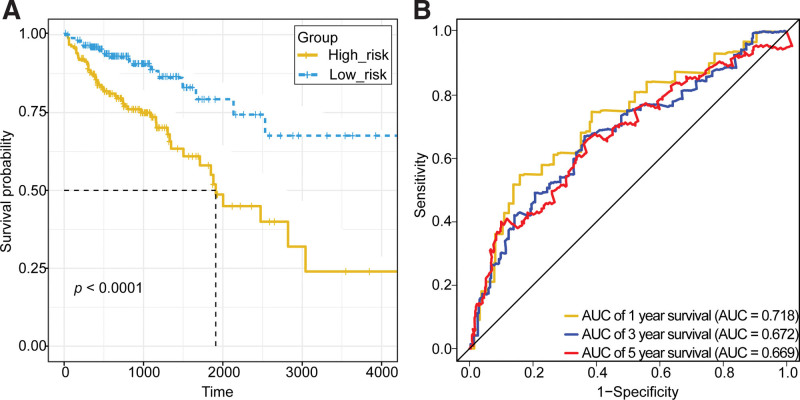
Survival analysis of 2 groups and ROC curves of the survival rate predicted by Riskscore in the TCGA dataset. AUC = area under curve.

The survival curve of the high-risk and low-risk groups showed a significant difference in survival status between the 2 groups (*P* < .0001) in the TCGA dataset, the survival rate was significantly higher in the low-risk group than it in the high-risk group (Fig. 5A). To assess the utility of the model, the area under the ROC curves (AUC) were obtained as 0.718, 0.672, and 0.669 based on the survival information of COAD patients at 1, 3, and 5 years in the TCGA dataset, respectively, showing that the prognostic model has certain predictive ability (Fig. 5B).

**Figure 6.**
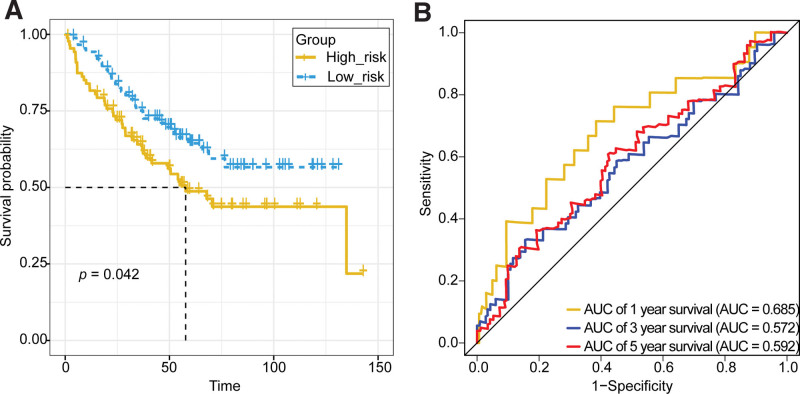
Survival analysis of 2 groups and ROC curves of the survival rate predicted by Riskscore in the GSE17536 dataset. AUC = area under curve.

The survival curve for the high- and low-risk groups in the external GSE17536 dataset also showed a significant difference in survival status between the 2 groups (*P* = .042), with a significantly higher survival rate in the low-risk group than it in the high-risk group (Fig. 6A). Similarly, the AUCs were obtained as 0.685, 0.572, and 0.592 based on the survival information of COAD patients at 1, 3, and 5 years in the GSE17536 dataset, respectively (Fig. 6B).

##### 3.4.2.2. Validation of the ability to predict metastasis

The ability of the prognostic Riskscore model to predict metastasis in COAD was evaluated using the GEO external datasets GSE41258 and GSE40367 (Table 2). The results showed that the sensitivities in the GSE41258 and GSE40367 datasets were 61.7% and 80%, respectively. Carcinoembryonic antigen (CEA) mRNA in draining venous blood is used in clinical testing. The abilities of CEA mRNA to predict CRC liver metastasis or local recurrence were reported, the sensitivities were 30% and 9%, and the ability of CEA levels to predict COAD recurrence was also reported, the sensitivity was 58.54%.^[44]^ Compared with CEA mRNA, the predictive ability of the Riskscore model is improved.

**Table 2:** Performance evaluation of prognostic models predicting COAD transfer based on GEO external datasets.

Dataset	SEN	SPE	ACC	PRE	F1	MCC	AUC
GSE41258	**0.6170**	0.3589	0.4837	0.4630	0.5273	−0.0278	0.4879
GSE40367	**0.8000**	0.6667	0.7500	0.8000	0.8000	0.4667	0.7333

SEN values of the prognostic Riskscore model were improved comparing with clinical used CEA are shown in bold.

ACC = accuracy, AUC = area under curve, COAD = colon adenocarcinoma, F1 = F-measure, GEO = gene expression omnibus, MCC = Matthews, PRE = precision, SEN = sensitive, SPE = specificity.

##### 3.4.2.3. Evaluation of whether Riskscore can be used as an independent prognostic factor

The prognostic model was used to determine the risk of COAD patients, it can be applied clinically only when it is an independent characteristic. Therefore, Cox regression analysis was used to test whether Riskscore can be an independent prognostic factor, simultaneously, several clinical characteristics (gender, age, and tumor stage) were also evaluated.

Both the TCGA dataset and the GSE17536 dataset showed that tumor stage and Riskscore could predict the prognosis of COAD patients independently (Tables 3 and 4).

**Table 3:** Cox analysis results from Risckscore combined with other clinical characteristics in the TCGA dataset.

Characteristics	Univariate cox analysis		Multivariate cox analysis	
	*P*	HR (95% CI)	*P*	HR (95% CI)
Tumor Stage	<.001	2.5 (1.9–3.3)	<.001	2.4 (1.8- 3.2)
Riskscore	<.001	2.7 (1.8–4)	.002	1.8 (1.2–2.6)
Age	.120	1 (1–1)	.018	1 (1–1)
Gender	.610	1.1 (0.71–1.8)	.650	0.9 (0.56–1.4)

CI = confidence interval, HR = hazard ratio, TCGA = The Cancer Genome Atlas.

**Table 4:** Cox analysis results from Riskscore combined with other clinical characteristics in the GEO dataset.

Characteristics	Univariate cox analysis		Multivariate cox analysis	
	*P*	HR (95% CI)	*P*	HR (95% CI)
Tumor Stage	<.001	2.9 (2.1–3.9)	<.001	3 (2.2–4.1)
Riskscore	.011	1.4 (1.1–1.7)	.011	1.2 (0.95–1.5)
Age	.490	1 (0.99–1)	.027	1 (1–1)
Gender	.670	1.1 (0.69–1.8)	.700	1.1 (0.67–1.8)

CI = confidence interval, GEO = Gene Expression Omnibus, HR = hazard ratio.

##### 3.4.2.4. Predictive probability of survival using a constructed nomogram

**Figure 7.**
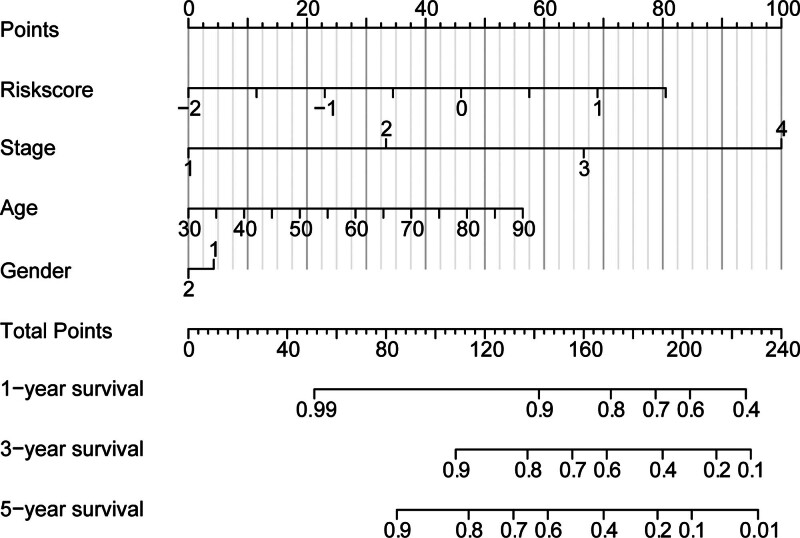
The nomogram was constructed by combining Riskscore with other clinical characteristics.

In addition, in clinical practice, the prognostic risk assessment of cancer patients is not just limited to “high” or “low,” sometimes, it is also necessary to estimate the survival rates by a combination of various clinical characteristics. A nomogram (Fig. 7) was constructed by combining Riskscore with clinical characteristics (gender, age, and stage), the probability of survival at 1, 3, and 5 years can be inferred based on the patient’s clinical information, providing a certain reference for subsequent clinical decisions.

**Figure 8.**
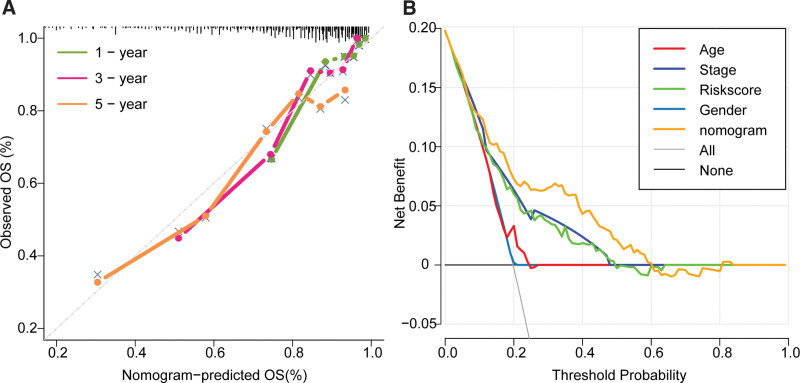
Evaluation of the predictive accuracy and clinical usefulness of the nomogram. (A) The calibration curve of the nomogram. (B) The decision curves. OS = overall survival.

Calibration curves were used to evaluate the predictive accuracies of the nomogram, the result showed that its C-index is 0.793, and there are no large deviations between predicted and actual performances as to the 1-year, 3-year, and 5-year survival rates, (Fig. 8A). The results of the decision curves showed that the net benefit of the nomogram was significantly superior to the net benefits of the other single variables when probabilities of death ranging is from 0.1 to 0.6 (Fig. 8B).

### 3.5. Analysis of the risk-related key pathways and tumor microenvironment

#### 3.5.1. Results of pathway enrichment analysis

GSEA pathway enrichment results showed that a total of 63 pathways were enriched in the high-risk group and 491 pathways were enriched in the low-risk group.

The results of pathway enrichment using the network propagation approach showed that 16,796 nodes, 504,027 edges, and 98 connected components were obtained by choosing the relationship pairs with action scores larger than 700 in the overall human PPI network. After removing duplicate action pairs, the maximum connected component contains a total of 16,566 nodes and 251,862 edges. Then the maximum connected component is used as the key subnet for subsequent analysis. Next, the pyroptosis genes were considered as the seeds to search the proximal genes, and the top 100 proximal genes were screened by influence scores. Finally, the seeds and their proximal genes were enriched in a total of 910 pathways.

**Figure 9.**
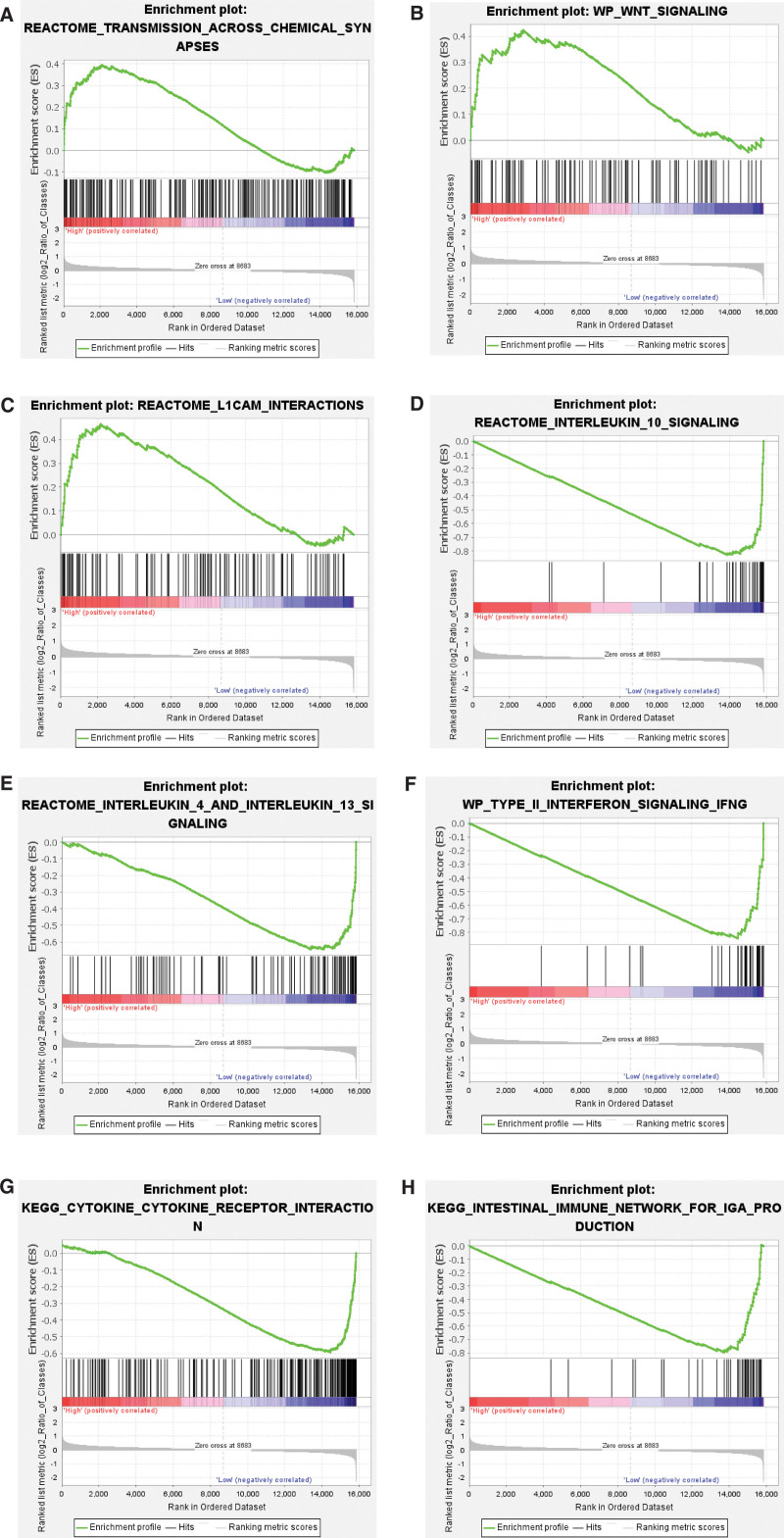
The GSEA results of the final 8 key pathways obtained from the pathway enrichment. (A–C) The GSEA results for the key pathways of High_risk group. (D–H) The GSEA results for the key pathways of Low_risk group. GSEA = gene set enrichment analysis.

The intersection of the above 2 methods showed that 3 pathways were enriched in the high-risk group, and a total of 282 pathways were enriched in the low-risk group. Further, in the low-risk group, the top 5 pathways associated with tumor development were selected based on the |NES| score. The GSEA results of the above 8 pathways were shown in Figure 9.

#### 3.5.2. Screening of the key genes and pathways

Due to the large number of genes involved in the resulting 8 pathways, the numbers of core genes for the 8 pathways were 51, 30, 29, 35, 49, 30, 88, and 24, respectively (Table S3, Supplemental Digital Content, http://links.lww.com/MD/N470 and Table S4, Supplemental Digital Content, http://links.lww.com/MD/N471, http://links.lww.com/MD/N471), univariate and multivariate Cox regression analyses of the core genes were performed with a threshold of *P* < .05. The results were shown in (Table 5) 11 important genes were obtained by the intersection of the results of the 2 regressions. Further, 6 final key genes were got with significant differences in expression (*P* < .05) and survival analysis (*P* < .05) between the high- and low-risk groups. Meantime, 6 pathways, in which the 6 key genes were located, were singled out (Table 6).

**Table 5:** Univariate cox regression and multivariable cox regression results of core genes.

Group	Pathway	Gene	Univariate cox		Multivariable cox	
			HR (95 CI)	*P*	HR (95 CI)	*P*
High risk	Transmission across chemical synapses pathway	ALDH5A1	4.1 (1.1–15)	.030	4.1 (1–16)	.049
		PPFIA4	8 (3.5–18)	<.001	6 (2.3–15)	.001
	Wnt signaling pathway	DVL3	20 (3.5–120)	.001	9 (1.4–60)	.023
		PLCB4	0.34 (0.12–0.94)	.037	0.29 (0.1–0.8)	.017
	L1CAM interactions pathway	VAV2	8.2 (1.7–41)	.010	9.8 (1.9–50)	.006
		NRCAM	4.2 (1.6–11)	.004	4.2 (1.6–11)	<.001
Low risk	IL-10 pathway	CCL22	0.3 (0.1–0.89)	.030	0.3 (0.1–0.89)	.030
	IL-4/IL-13 pathway	RORC	0.21 (0.08–0.55)	.001	0.25 (0.094–0.66)	.005
	Intestinal immune network for IgA production pathway	TNFRSF17	0.26 (0.1–0.68)	.006	0.32 (0.12–0.89)	.028
	Cytokine-cytokine receptor interaction pathway	CCL24	0.27 (0.088–0.82)	.020	0.29(0.093–0.92)	.035
	IFNγ pathway	PSMB9	0.29 (0.089–0.97)	.045	0.29 (0.089–0.97)	.045

ALDH5A1 = Aldehyde Dehydrogenase 5 Family Member A1, CCL22 = C-C Motif Chemokine Ligand 22, CCL24 = C-C Motif Chemokine Ligand 22, CI = confidence interval, DVL3 = disheveled segment polarity protein 3, HR = hazard ratio, NRCAM = neuronal cell adhesion molecule, PLCB4 = Phospholipase C Beta 4, PPFIA4 = PTPRF Interacting Protein Alpha 4, PSMB9 = proteasome 20S subunit beta 9, RORC = RAR related orphan receptor C, TNFRSF17 = TNF receptor superfamily member 17, VAV2 = Vav Guanine Nucleotide Exchange Factor 2.

**Table 6:** Expression levels and survival analysis of key genes in high and low risk groups.

Group	Pathway		Gene	*P*	
				Expression	KM
High-risk	Transmission across chemical synapses pathway		ALDH5A1	.001	.030
	Wnt signaling pathway	Wnt signaling pathway	DVL3	<.001	.031
		L1CAM interactions pathway	NRCAM	.014	.001
Low-risk	IL-4/IL-13 pathway	IL-4/IL-13 pathway	RORC	.036	.037
		IFNγ pathway	PSMB9	<.001	.015
	intestinal immune network for IgA production pathway		TNFRSF17	.008	.003

ALDH5A1 = aldehyde dehydrogenase 5 family member A1, DVL3 = disheveled segment polarity protein 3, KM = Kaplan-Maeier, NRCAM = neuronal cell adhesion molecule, PSMB9 = proteasome 20S subunit beta 9, RORC = RAR related orphan receptor C, TNFRSF17 = TNF receptor superfamily member 17.

Three of these pathways were specifically up-regulated in the high-risk group, they were the transmission across chemical synapses pathway, the Wnt signaling pathway, and the L1CAM interactions pathway. The key genes in the high-risk group were aldehyde dehydrogenase 5 family member A1 (*IL*), disheveled segment polarity protein 3 (*DVL3*), and neuronal cell adhesion molecule *(NRCAM*). Since *NRCAM*, a key gene in the L1CAM interactions pathway, was the downstream target gene of the Wnt signaling pathway, so the 2 pathways were merged as one pathway for subsequent analysis.

The 3 other pathways were specifically up-regulated in the low-risk group, they were the IL-4/IL-13 pathway, the intestinal immune network for immunoglobulin A (IgA) production pathway, and the IFNγ pathway. Since the IFNγ pathway was activated by the downstream factors of the IL-4/IL-13 pathway, they were analyzed together as one pathway. The key genes in the low-risk group were RAR-related orphan receptor C (*RORC*), proteasome 20S subunit beta 9 (*PSMB9*), and TNF receptor superfamily member 17 (*TNFRSF17*). In summary, 2 key pathways were finally up-regulated in the high-risk group, and 2 pathways were up-regulated in the low-risk group. A total of 4 key pathways were analyzed for subsequent analysis.

### 3.6. Mechanism analysis of key pathways associated with high and low risk

#### 3.6.1. High-risk group: transmission across chemical synapses pathway, key gene: ALDH5A1

As to key gene *ALDH5A1*, existing studies have found that *ALDH5A1* was regulated by hepatocyte nuclear factor 4 alpha in renal cell carcinoma, and *ALDH5A1* was up-regulated in COAD stem cells, except for these, there was little insight into the regulation mechanism of *ALDH5A1* in tumors, and there were no studies that revealed the relationship of *ALDH5A1* with COAD, or its key role in COAD progression at the molecular level.^[45]^

*ALDH5A1* was a member of the Aldehyde dehydrogenase (ALDH) family, encoding Succinate semialdehyde dehydrogenase, and Succinate semialdehyde dehydrogenase was mainly involved in the degradation of Gamma-Aminobutyric acid (GABA) in transmission across chemical synapses pathway. It has been demonstrated that GABA could accumulate abnormally in COAD. The degradation of GABA could produce succinic acid.^[46]^

**Figure 10.**
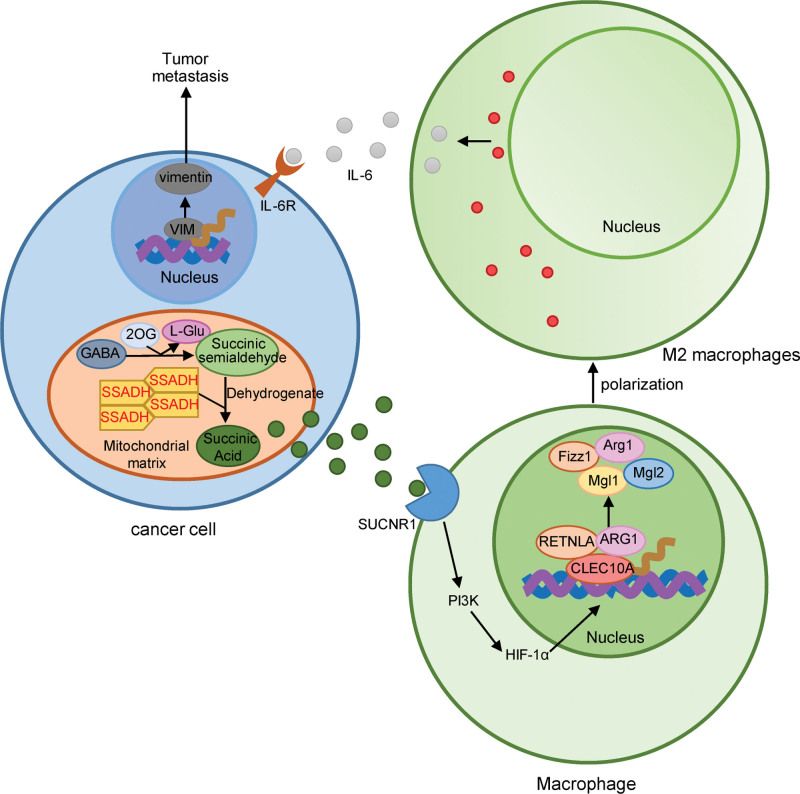
Mechanism of L1CAM interactions pathway involved with ALDH5A1. GABA = Gamma-Aminobutyric acid, IL = aldehyde dehydrogenase 5 family member A1, SSADH = Succinate semialdehyde dehydrogenase, hypoxia-inducible factor-1α.

In this study, *ALDH5A1* was found to be the key COAD-promoting gene involved in the transmission across chemical synapses pathway in the high-risk group. We proposed that the mechanism leading to the high risk of COAD is most likely that the high expression of *ALDH5A1* results in the yielding of high concentrations of succinic acid by degradation of GABA in COAD (Fig. 10). High concentrations of succinic acid could combine with the activated succinate receptor SUCNR1, thereby, activating downstream hypoxia-inducible factor-1α via the PI3K/AKT pathway. Then, HIF-1 polarized macrophages to the M2 phenotype, which secreted tumor-promoting factors (e.g., IL-6) and promoted tumor growth and metastasis.^[47]^ *ALDH5A1* had been detected as over-expressed in several other tumor tissues,^[48,49]^ and some studies had also confirmed that the ALDH gene family was over-expressed in COAD stem cells and then initiated COAD tumorigenesis.^[45]^ Increased concentrations of succinic acid were found in a variety of tumors, including COAD.^[50,51]^ These previous studies further confirmed the reliability of the mechanism proposed in this study.

**Figure 11.**
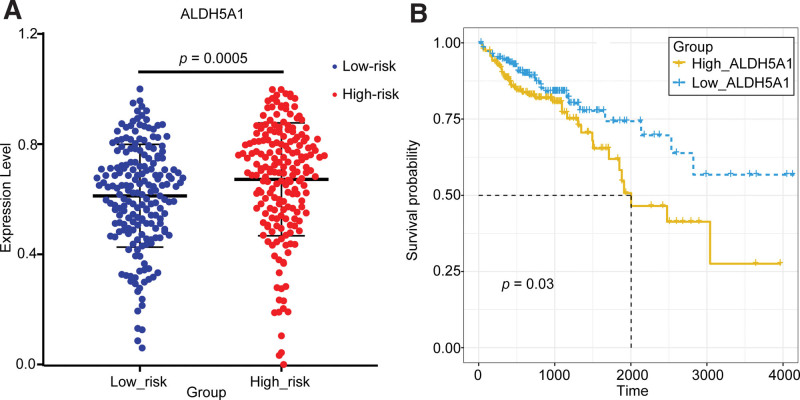
Validation of the key gene *ALDH5A1* in the TCGA dataset. (A) The expression validation of *ALDH5A1* in the high-risk group and low-risk group. (B) The survival analysis of ALDH5A1 in the high-express group and low-express group. ALDH = aldehyde dehydrogenase.

**Figure 12.**
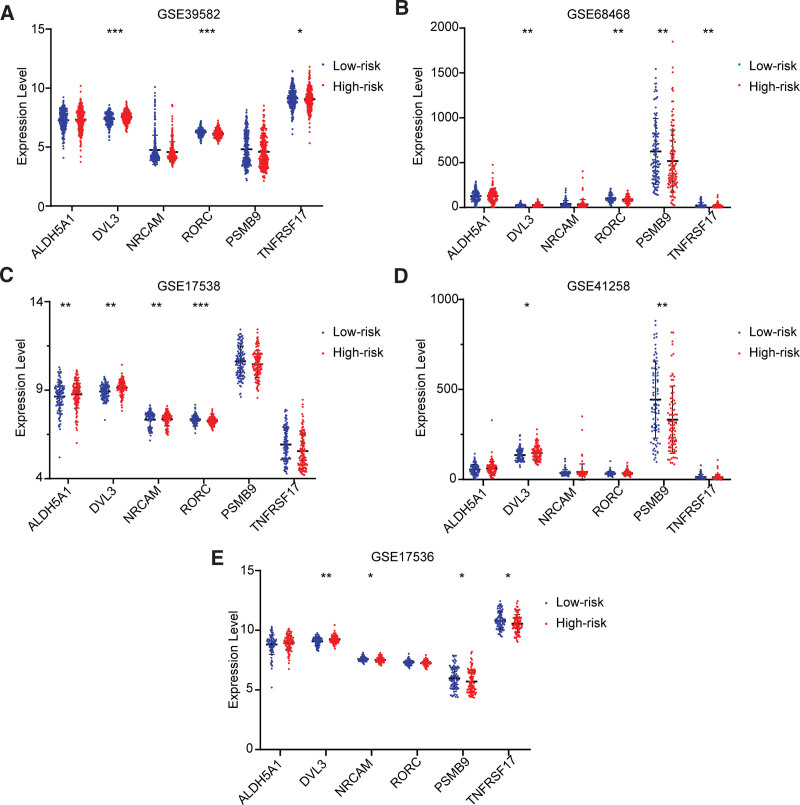
The expression validation results of the key genes in the high-risk group and low-risk group based on the GEO external datasets. ALDH = aldehyde dehydrogenase, DVL3 = disheveled segment polarity protein 3, NRCAM = neuronal cell adhesion molecule, PSMB9 = proteasome 20S subunit beta 9, RORC = RAR-related orphan receptor C, TNFRSF17 = TNF receptor superfamily member 17.

The relationship between the key gene *ALDH5A1* and the risk of COAD was validated by the expression and survival curve analysis (Fig. 11). As seen in Figure 11, the expression of *ALDH5A1* was significantly different between the high-risk group and low-risk group and was up-regulated in the high-risk group. The survival analysis curve showed that the survival rate of the *ALDH5A1*-high-express group was significantly lower than that of the *ALDH5A1*-low-express group. In a word, the expression of *ALDH5A1* was significantly up-regulated and the survival rate was significantly lower in the high-risk group. High expression of *ALDH5A1* in the high-risk group was also validated in the GSE17538 dataset, where *ALDH5A1* was significantly differentially expressed in 2 groups (*P* = .0006), and it was up-regulated in the high-risk group (Fig. 12).

#### 3.6.2. High-risk group: Wnt signaling pathway, key genes: *DVL3* and *NRCAM*

**Figure 13.**
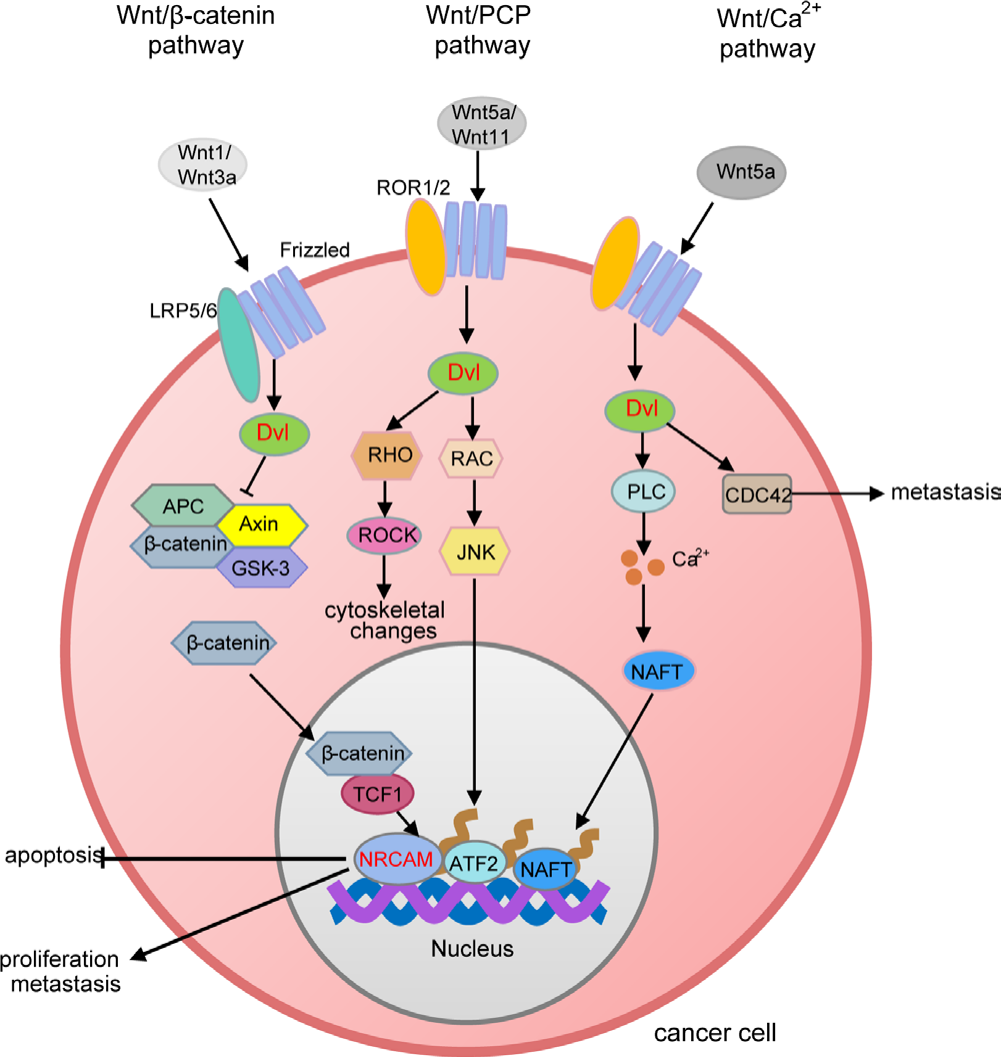
Mechanism of Wnt signaling pathway involved with *DVL3* and *NRCAM*. DVL3 = disheveled segment polarity protein 3, JNK = c-Jun N-terminal kinase, NRCAM = neuronal cell adhesion molecule, PLC = phospholipase C, TCF = T-cell factors.

In this study, *DVL3* and *NRCAM* were found to be the key COAD-promoting genes involved in the Wnt signaling pathway in the high-risk group. We proposed a mechanism in which the up-regulation of *DVL3* eventually activated the target gene *NRCAM*, which may promote cancer cell proliferation and metastasis (Fig. 13).

The overexpression of *DVL3* in COAD has been acknowledged in several studies. Velázquez et al^[52]^ found that *DVL3* was significantly over-expressed in the SW480 COAD cell line compared with nonmalignant cells of the intestine by protein blotting. Ali et al^[53]^ discovered that using butyrate and knocking out *DVL3* could significantly reduce the proliferation of cancer cells by cell transfection experiments, suggesting that the anti-cancer molecule butyrate inhibited the Wnt signaling pathway and resisted COAD by inhibiting *DVL3*. Esulifa et al^[54]^ revealed that the over-expressed Rac1b in COAD up-regulated the Wnt signaling pathway and promoted COAD in recombination experiments. These studies further demonstrated the reliability of the mechanism proposed in this study, that is the up-regulation of *DVL3* could promote COAD through the Wnt signaling pathway in the high-risk group. The up-regulation of *NRCAM* in COAD cells has also been demonstrated in many studies. Conacci-Sorrell et al^[55]^ found by qRT-PCR that *NRCAM* was significantly over-expressed in the human COAD cell lines SW48, SW480, and HCT116, whereas it could not be detected in normal colon tissue samples. These studies further demonstrate the reliability of the mechanism proposed in this study, that is the up-regulation of *NRCAM* could promote COAD in the high-risk group.

Up-regulation of the Wnt signaling pathway had been proposed as a marker sign for COAD development.^[56]^ The Wnt signaling pathway is divided into classical and non-classical pathways. In the classical Wnt/β-catenin pathway, the ligands Wnt1 and Wnt3abind to membrane receptors to recruit *DVL3*, subsequently, the degradation of β-catenin mediated by adenomatous polyposis coli or axis inhibited, and free β-catenin in the cytoplasm is stabilized. Next, the β-catenin protein translocates to the nucleus, where it interacts with T-cell factors (TCFs) to regulate gene expressions, such as *MYC* and *NRCAM*, to accelerate the cell cycle and promote cancer cell metastasis. In the non-classical Wnt/PCP pathway, Wnt5a/Wnt11 triggers the activation of c-Jun N-terminal kinase (JNK) and regulates cytoskeletal rearrangement and cell migration by recruiting *DVL3* to interact with the small GTPases RHOA and Rac. In the non-classical Wnt/Ca2 + pathway, the ligand Wnt5a induces *DVL3* hyperphosphorylation, which promotes Ca^2+^ inward flow and cell migration by activating Phospholipase C.^[51,57]^ All the above suggests that up-regulation of *DVL3* in the classical and non-classical Wnt signaling pathways can promote COAD. The key gene *NRCAM* is one of the most widely targeted genes of the Wnt/β-catenin pathway, up-regulation of *NRCAM* could protect COAD cells from apoptosis by activating extracellular signal-regulated kinases and AKT signaling pathways,^[58]^ and up-regulation of *NRCAM* also can lead to tumorigenesis, cell proliferation, and migration.^[55]^ Therefore, it can be speculated that the over-expression of *NRCAM* plays a promoting role in COAD development.

**Figure 14.**
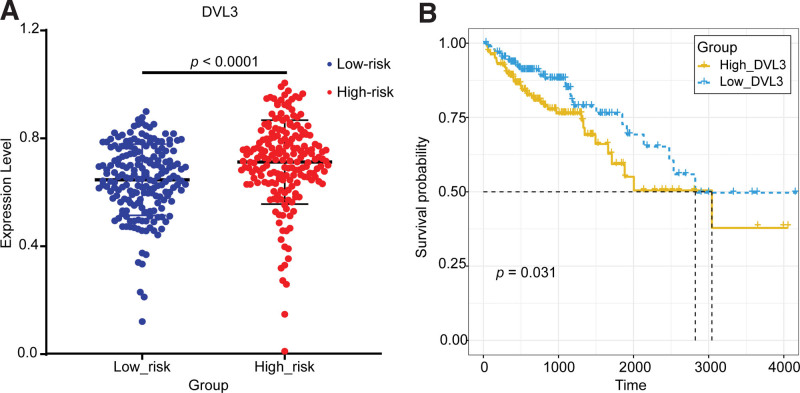
Validation of the key gene *DVL3* in the TCGA dataset. (A) The expression validation of DVL3 in the high-risk group and low-risk group. (B) The survival analysis of DVL3 in the high-express group and low-express group. DVL3 = disheveled segment polarity protein 3, TCGA = The Cancer Genome Atlas.

The relationship between the key gene *DVL3* and the risk of COAD was validated by the expression and survival curve analysis (Fig. 14). As seen in Figure 14, the expression of *DVL3* was significantly different between the high-risk group and low-risk group and was up-regulated in the high-risk group. The survival analysis curve showed that the survival rate of the *DVL3*-high-express group was significantly lower than that of the *DVL3*-low-express group. In one word, the expression of *DVL3* was significantly up-regulated and the survival rate was significantly lower in the high-risk group. High expression of *DVL3* in the high-risk group was also validated in 5 GEO datasets (GSE39582, GSE68468, GSE17538, GSE41258, and GSE17536), where *DVL3* was significantly differentially expressed in 2 groups (*P* < .0001, *P* = .0088, *P* = .0002, *P* = .0455, and *P* = .0050), and it was up-regulated in the high-risk group (Fig. 12).

**Figure 15.**
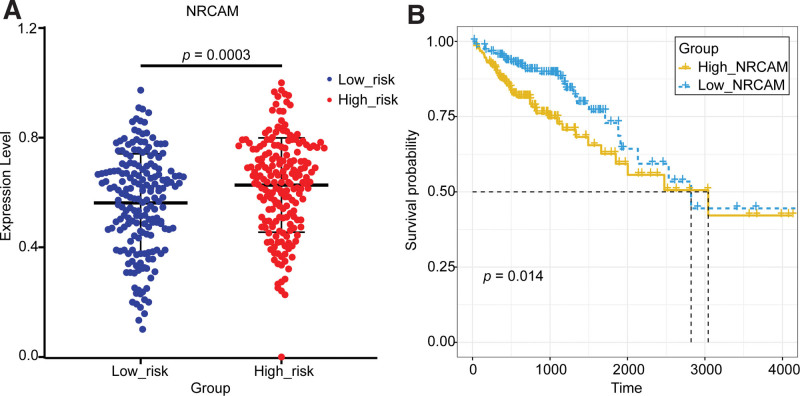
Validation of the key gene *NRCAM* in the TCGA dataset. (A) The expression validation of NRCAM in the high-risk group and low-risk group. (B) The survival analysis of NRCAM in the high-express group and low-express group. NRCAM = neuronal cell adhesion molecule, TCGA = The Cancer Genome Atlas.

The relationship between the key gene *NRCAM* and the risk of COAD was validated by the expression and survival curve analysis (Fig. 15). As seen in Figure 15, the expression of *NRCAM* was significantly different between the high-risk group and low-risk groups and was up-regulated in the high-risk group. The survival analysis curve showed that the survival rate of the *NRCAM*-high-express group was significantly lower than that of the *NRCAM*-low-express group. This was also validated in the GSE17538(*P* = .0025), where *NRCAM* was significantly up-regulated in the high-risk group (Fig. 12).

#### 3.6.3. Low-risk group: IL-4/IL-13 pathway, key genes: *RORC* and *PSMB9*

In this study, *RORC* and *PSMB9* were found to be the key COAD-suppressing gene involved in the IL-4/IL-13 pathway in the low-risk group. Several existing studies suggested that up-regulation of the IL-4/IL-13 pathway inhibited the development of COAD. An immunogen is to the chemical study of COAD patients showed that patients with high-expression of IL-13R had fewer lymph node metastases. Scrum levels of IL-13 were significantly lower in COAD patients in an advanced stage, and this was associated with a poorer prognosis.^[59]^ These studies confirmed that up-regulated IL-4/IL-13 pathway inevitably suppressed the progression of COAD.

**Figure 16.**
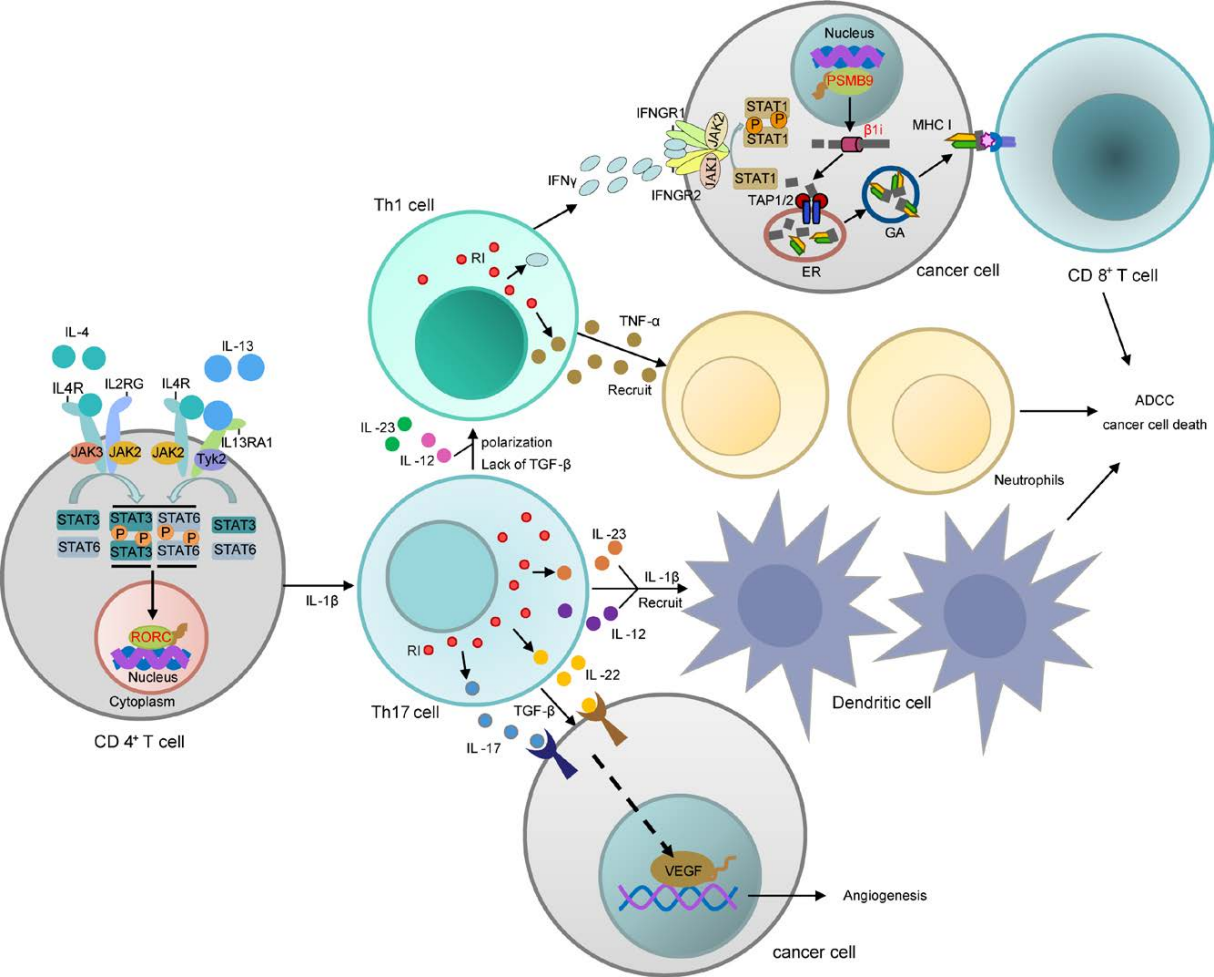
Mechanism of IL-4/IL-13 pathway involved with *RORC* and *PSMB9*. IL = aldehyde dehydrogenase 5 family member A1, PSMB9 = proteasome 20S subunit beta 9, RORC = RAR-related orphan receptor C.

As for the key gene *RORC*, the mechanism leading to a low risk of COAD may be that the over-expression of the target gene *RORC,* which is located the downstream of IL-4/IL-13 pathway, can cause primitive CD4^+^ T cells to polarize into the Th17 cells, then the Th17 cell secretes IL-17 and IFNγ (Fig. 16). It has been demonstrated that the main Th17 cell effector cytokines, IL-17, can up-regulate lymphocyte proliferation and down-regulate immunosuppressive Treg and Th2 cells, finally exerting anti-tumor effects to inhibit tumor growth.^[60]^ The anti-COAD effect of *RORC* had been demonstrated in several studies. Pan et al revealed that the expression of *RORC* was reduced in COAD tissues and COAD patients compared with normal tissues using real-time quantitative PCR, and the down-regulation expression of *RORC* was accompanied by liver metastases.^[61]^ Hu et al^[62]^ proved by cell culture that *RORC* agonists could enhance Th17 cell differentiation, migration, and infiltration, inhibits TGF and Treg cell differentiation, and increase antitumor effector T cell function. These existing studies further confirmed the reliability of the finding that over-expression of the key gene *RORC* in the low-risk group suppressed COAD.

**Figure 17.**
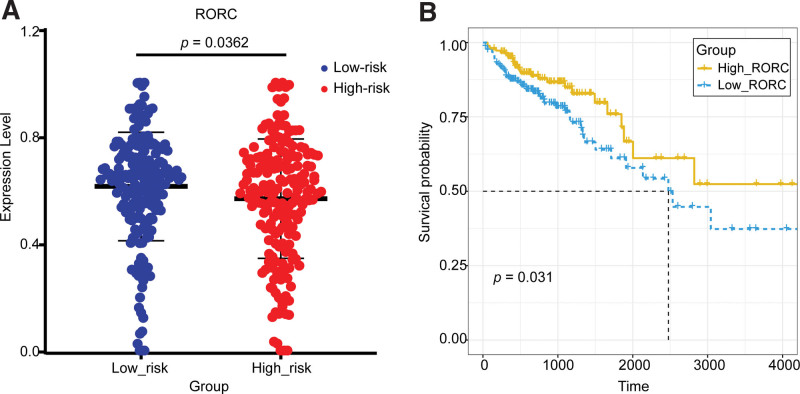
Validation of the key gene *RORC* in the TCGA dataset. (A) The expression validation of RORC in the high-risk group and low-risk group. (B) The survival analysis of RORC in the high-express and low-express groups. RORC = RAR-related orphan receptor C, TCGA = The Cancer Genome Atlas.

The relationship between the key gene *RORC* and the risk of COAD was validated by the expression and survival curve analysis (Fig. 17). As seen in Figure 17, the expression of *RORC* was significantly up-regulated in the low-risk group, and the survival rate of the *RORC*-high-express group was significantly higher than that of the low-express group. This was also validated in 3 GEO datasets (GSE39582, GSE68468, and GSE17538) (Fig. 12), where *RORC* was significantly up-regulated in the low-risk group (*P* < .0001, *P* = .0016, and *P* < .0001).

However, we noted that several studies had come to the opposite conclusion. A study suggested that the over-expression of *RORC* would promote Th17-mediated COAD.^[63]^ Using the immunohistochemistry method, Yoshida et al found that a high *RORC*/CD3 ratio was significantly associated with lymph node metastasis and a poor prognosis.^[64]^

The root causes of the contrary conclusion were further explored here. Th17 cells were plastic, environmental factors could determine that Th17 cells transformed into- or anti-cancer phenotype.^[65]^

**Figure 18.**
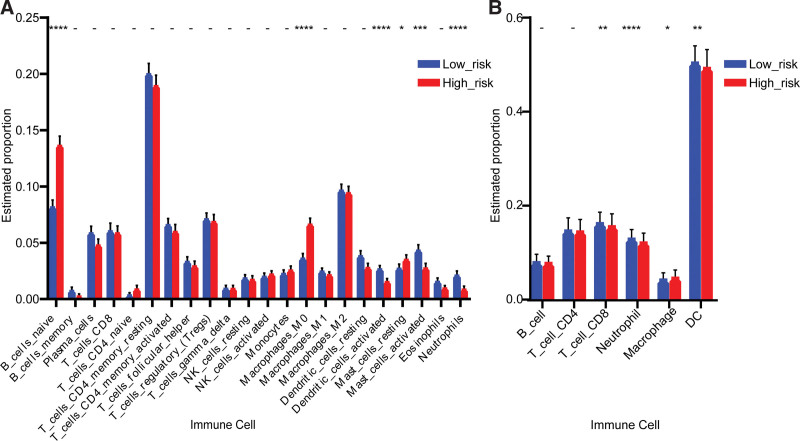
Results of the immune infiltration in the high-risk group and low-risk group. (A) The infiltration degree of the 22 immune cells obtained by the CIBERSORT algorithm. (B) The infiltration degree of 6 immune cells obtained by the TIMER algorithm.

It had been proved that when TGF-β was present, Th17 cells played a pro-cancer role by secreting IL-17 and IL-22, which would result in angiogenesis and immunosuppression.^[66]^ However, the presence of IL-1β promoted the differentiation and activation of Th17 cells, then the Th17 cells migrated to tumor tissues with the help of cell adhesion molecules.^[67]^ Th17 cells could indirectly reduce tumor growth by inducing CCL2 and CCL20 expression, which consequently promotes the activation and recruitment of DCs, CD8^+^ T cells, and CD4^+^ T cells in the tumor tissues.^[68]^ We did find out by immune infiltration analysis that DCs were infiltrated significantly more in the low-risk group compared with the high-risk group, suggesting that DCs with a high degree of infiltration could inhibit the progression of COAD (Fig. 18). There were several studies confirmed the anti-COAD mechanism of DCs. DCs can present antigens to CD8^+^ T cells, or secrete IL-12 to activate killer T cells, then result in clearing cancer cells, meanwhile, IFNγ secreted by activated killer T cells promote DCs maturation through a positive feedback regulation.^[69]^ Currently, DCs are extensively studied in anti-cancer vaccine development.^[70]^ The above proved the reliability that high-expression of the key gene *RORC* could suppress the COAD by recruiting DCs through Th17 cells in the low-risk group when environmental factors IL-1β were present.

Th17 cells could be converted to Th1 cells induced by IL-12 and IL-23 with reduced lymphocytes,^[71]^ or in the presence of little TGF-β or no TGF-β at all in the environment. Then Th1 cells released TNF-α and Interferon gamma (IFNγ) which act as cancer suppressors. Several in vitro experiments had shown that the main effectors of the Th1 phenotype were TNF-α and IFNγ.^[65]^

**Figure 19.**
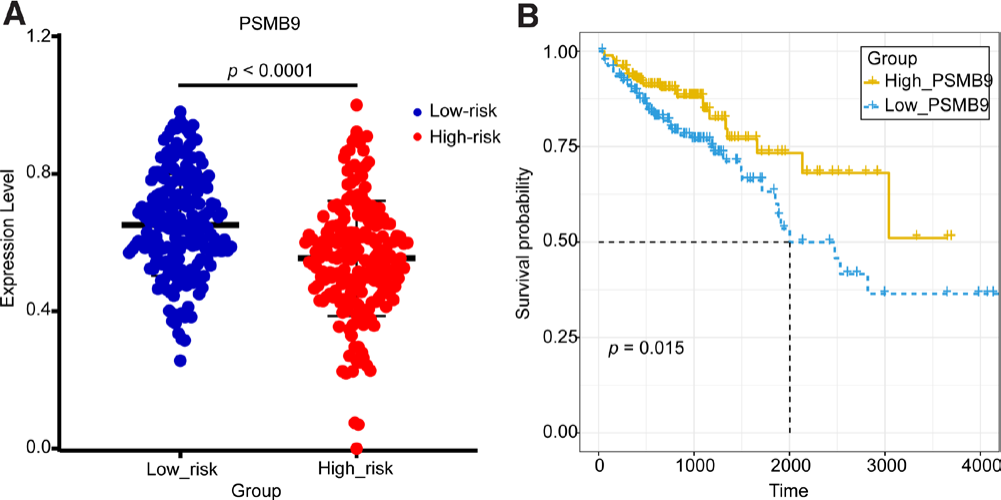
Validation of the key gene *PSMB9* in the TCGA dataset. (A) The expression validation of PSMB9 in the high-risk group and low-risk group. (B) The survival analysis of PSMB9 in the high-express group and low-express group. PSMB9 = proteasome 20S subunit beta 9, TCGA = The Cancer Genome Atlas.

TNF-α could kill tumor cells and stimulate neutrophil recruitment.^[72]^ And then under conditions without TGF-β, neutrophils could secret cytokines TNF-α and express T cell receptor (TCR), recruit CD8^+^ T cells, and enhance antibody-dependent cell-mediated cytotoxicity, ultimately inhibiting tumor growth.^[73]^ We did find out by immune infiltration analysis that neutrophils were infiltrated significantly more in the low-risk group compared with the high-risk group (Fig. 19). The result was consistent with the above anti-tumor mechanism. Several studies had revealed the anti-tumor effects of neutrophils. Geh et al illustrated that neutrophils with a dual role would transform to the anti-tumor phenotype N1 when TGF-β was suppressed.^[74]^ Linde et al^[72]^ eradicated solid and metastatic tumors by stimulating neutrophil activation, recruitment, and proliferation using neutrophil activation therapy, which was composed of tumor necrosis factor (TNF), anti-CD40 monoclonal antibody, and tumor-specific binding antibody. The study indicated that activated neutrophils could exert antitumor activity. In conclusion, the above indicated that in the low-risk group, when TGF-β was deficient, Th17 cells could convert to Th1 cells and release TNF-α, which in turn recruit neutrophils and ultimately have anti-cancer effects mechanistically.

The other main effector of the Th1 is IFNγ. IFNγ up-regulated a set of interferon-stimulated genes involved in immune activation, thereby suppressing tumor growth.^[60]^ The tumor suppressor gene IRF1 activated by IFNγ could activate key gene *PSMB9*.^[12–15,75]^ *PSMB9* encoded a 20S core β subunit which participated in the formation of the immunoprotein some β1i. The immunoprotein then processed tumor antigens to antigenic peptides, which were presented to the surface of cancer cells for recognition and destruction by CD8^+^ T cells^[76]^ (Fig. 16). The anti-COAD effects of IFNγ had been confirmed by a lot of studies. Geller et al demonstrated that IFNγ increased proteasome activity in the HT29 cell line and promoted apoptosis of COAD cells.^[77]^ Yao et al^[78]^ examined circulating-peripheral blood samples from COAD patients and found that IFNγ tended to decrease with the progression of COAD. As for the key gene *PSMB9*, its anti-tumor effects had also been confirmed in some studies. Siebenkäs et al^[79]^ found that *PSMB9* was significantly down-regulated in 8 COAD cell lines compared with normal colon cell lines by qRT-PCR. Imanishi et al^[80]^ revealed that 4 out of 5 COAD cell lines showed a significant decrease in gene and protein expression of *PSMB9* by qRT-PCR and Western blot. In summary, in the low-risk group, when TGF-β was lacking, Th17 cells could convert into Th1 cells and release IFNγ, further activating *PSMB9*, which composes the immunoproteasome. The immunoproteasome helped complete antigen presentation and triggered the recognition and destruction of cancer cells by CD8^+^ T cells.

In this study, *PSMB9* was identified as the key COAD-suppressing gene in the low-risk group. The expression and survival curve analysis of *PSMB9* also validated its suppressive effect on COAD (Fig. 19). The expression of *PSMB9* was significantly different between the high-risk group and the low-risk group and was higher in the low-risk group compared with the high-risk group. The survival curve showed that the survival rate was significantly higher in the *PSMB9*-high-express group than in the *PSMB9*-low-express group (Fig. 12). That means the expression of *PSMB9 was* significantly up-regulated and the survival rate was significantly higher in the low-risk group. High expression of *PSMB9* in the low-risk group was also validated in 3 GEO datasets (GSE68468, GSE41258, and GSE17536), where *PSMB9* was significantly up-regulated in the low-risk groups (*P* = .0091, *P* = .0001, and *P* = .0278), and it was up-regulated in the low-risk group. The results were consistent with the findings of the study.

#### 3.6.4. Low-risk group: intestinal immune network for IgA production pathway, key genes: *TNFRSF17*

In this study, *TNFRSF17* was identified as the key COAD-suppressing gene involved in the intestinal immune network for the IgA production pathway in the low-risk group.

It had been proved that over-expression of *TNFRSF17* could resist COAD,^[81]^ however, the specific mechanism of *TNFRSF17* in COAD had not been reported. *TNFRSF17* encoded the BCMA protein, which was involved in the activation of B1 cells, then the B1 cells go on to form IgA + plasma cells which can synthesize and transport IgA^[82]^ (Fig. 19). The anti-COAD effect of IgA had been revealed in several studies. A study on COAD patients vaccinated with CEA antigens indicated that IgA could kill COAD cells using antibody-dependent cell-mediated cytotoxicity and Complement-dependent cytotoxicity, and overall survival was significantly longer in patients with high IgA levels.^[83]^ Similar findings had been obtained in other studies.^[84]^ Li et al^[85]^ showed that flavoprotein polysaccharide (P1) suppressed COAD by enhancing mucosal IgA production throughacyto-inhibition test. Thus, we hypo the sized that the low risk mechanism related to *TNFRSF17* may be that the up-regulation of *TNFRSF17* promotes IgA secretion, which in turn mediates the killing of COAD cells through cytotoxicity, finally inhibiting their proliferation and metastasis.

The over-expression of *TNFRSF17* in COAD had been confirmed in several studies. Song et al found that the expression of *TNFRSF17* was significantly lower in COAD cell lines than that in healthy colon tissues by quantitative qRT-PCR.^[81]^ Moreover, the COAD cells with up-regulated *TNFRSF17* had a significantly lower capacity for proliferation, invasion, and migration than control cells.

**Figure 20.**
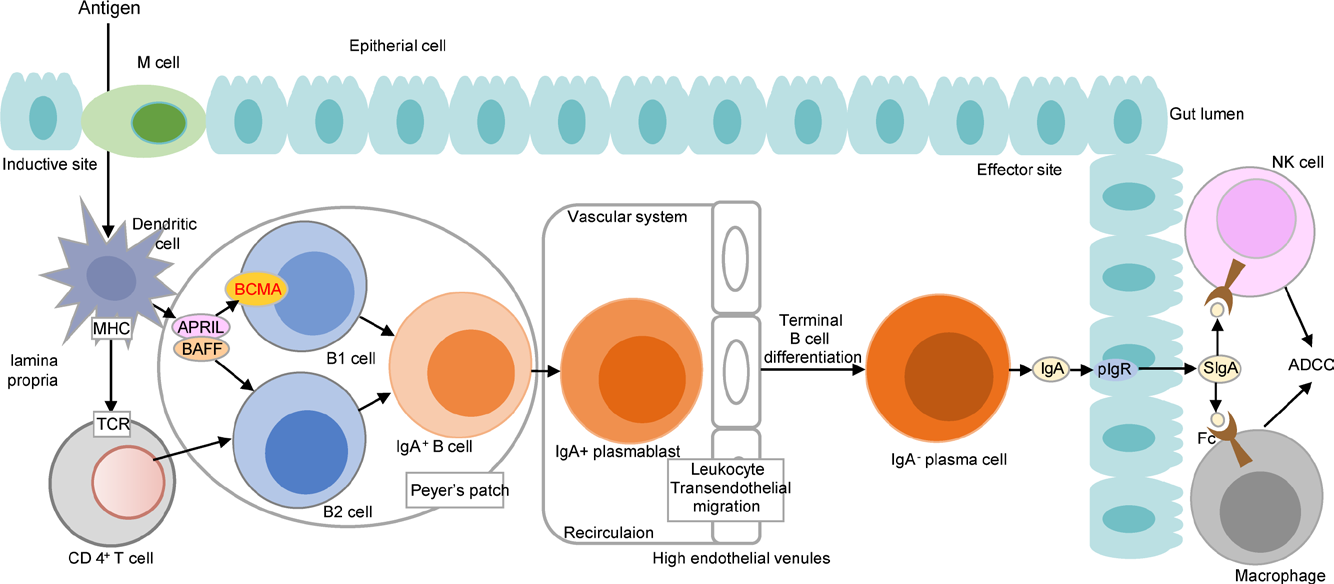
Mechanism of intestinal immune network for IgA production pathway involved with *TNFRSF17*. TNFRSF17 = TNF receptor superfamily member 17. TCR = TNF-α and express T cell receptor.

**Figure 21.**
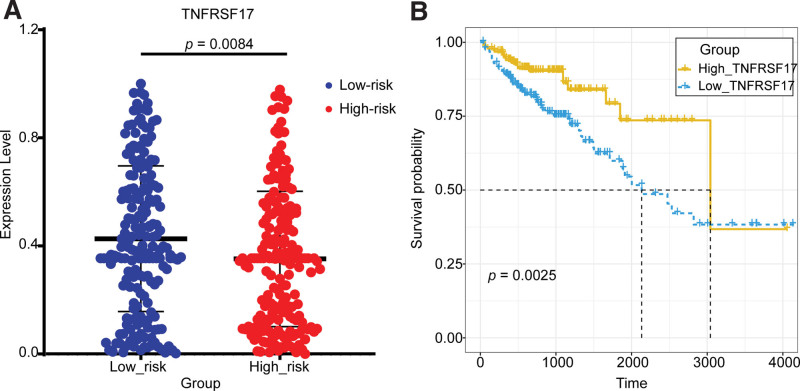
Validation of the key gene *TNFRSF17* in the TCGA dataset. (A) The expression validation of *TNFRSF17* in the high-risk group and low-risk group. (B) The survival analysis of *TNFRSF17* in the high-express group and low-express group. TNFRSF17 = TNF receptor superfamily member 17, TCGA = The Cancer Genome Atlas.

The relationship between the key gene *TNFRSF17* and the risk of COAD was validated by the expression and survival curve analysis. There was a significant difference in *TNFRSF17* expression between the low-risk group and the high-risk group, with significant over-expression in the low-risk group (Fig. 20). Survival analysis curves showed that the survival rate was significantly higher in the *TNFRSF17*-high-express group than that in another group (Fig. 21). In a word, the expression of *TNFRSF17* was significantly up-regulated and the survival rate was significantly lower in the low-risk group. High expression of *TNFRSF17* in the low-risk group was also validated in 3 GEO datasets, GSE39582, GSE68468, and GSE17536 (*P* = .0363, *P* = .0037, and *P* = .0260), and it was significantly up-regulated in the low-risk group (Fig. 11).

## 4. Discussion

Our study tried to find a prognostic model for COAD based on pyroptosis-related genes and to reveal mechanisms associated with poor prognosis, which will be helpful for precision therapy. However, in order to improve the clinical cure rate of COAD, experts around the world are also actively exploring better treatment strategies. Targeted therapy based on molecular markers has led to a significant improvement in the overall prognosis of patients with advanced COAD. In our study, 6 key genes, *ALDH5A1, DVL3, NRCAM, RORC, PSMB9,* and *TNFRSF17*, which play key roles in COAD, were identified, while the potential of these genes as drug targets needs to be further examined.

Among the 6 key genes, 2 of them have been confirmed as drug targets for COAD. *NRCAM* had been proposed in previous studies as an independent marker of poor prognosis in advanced COAD using immunohistochemical experiments and tissue microarrays methods, and its over-expression had been found to inhibit the effect of chemotherapy with 5-fluorouracil. As for gene *RORC*, its agonists were considered to have great anti-cancer potential. The studies developing single or combination immunotherapies based on *RORC* confirmed the immune activation and anti-COAD effects of its agonists. For example, *RORC* agonists combined with the checkpoint inhibitor anti-CTLA-4 could enhance Th17 cell differentiation, migration, and infiltration, inhibit Treg cell production, increase the function of anti-tumor effector T cells, and inhibit the growth of MC38 COAD cells. Currently, several clinical trials based on *RORC* agonists are exploring their safety and efficacy. Other 2 key genes had been researched for drug targets in other oncology areas. For example, the gene *TNFRSF17* had been used as a drug target for multiple myeloma, the drug Belantamab Mafodotin targeted was approved by the FDA in 2020.^[86]^ Gene *ALDH5A1* has been suggested as a poor prognostic marker for papillary thyroid carcinoma and a new potential target for human breast ductal carcinoma by gene knock-out, cell proliferation, Transwell migration/invasion assay, wound healing, and qRT-PCR methods. All of these indicated the importance of these key genes and also proved their potential as a drug target for COAD.

TME plays a critical role in tumorigenesis, tumor invasion, and metastasis. Different TMEs lead to different outcomes, just as the key gene RORC, our study elucidated that the over-expression of RORC triggered complex immune microenvironment changes in the presence of different immune factors. Over-expression of *RORC* resulting CD4 + T cells polarizing into Th17 cells, while Th17 cells were plastic, environmental factors could determine that Th17 cells transformed intopro- or anti-cancer phenotype. When TGF-β was present, Th17 cells played a pro-cancer role by secreting IL-17 and IL-22, while when IL-1βwas present, Th17 inducing CCL2 and CCL20 expression, DCs, and other immune cells were activated and recruited, resulting the inhibition of COAD. Similarly, when TGF-β was absent, Th17 could convert to Th1 cells, causing neutrophil recruitment or destruction of cancer cells by CD8 + T cells, resulting the inhibited tumor growth. It can be seen that the tumor immune microenvironment is a determinant of response to cancer. Some studies had proposed reshaping the tumor immune microenvironment to enhance immunotherapy response.^[87,88]^ Clinically, ICIs have been combined with chemotherapy drugs to enhance immunity and kill cancer cells at the same time.^[89–92]^ Remodeling tumor immune microenvironment has great prospects for cancer treatment. DCs and neutrophils activations have been proposed as anti-cancer strategies.^[70,72]^ The plastic of Th17 illustrated in this study showed that enhanced activations of DCs and neutrophils may inhibit COAD.

This study is based on the TCGA dataset, although it has been validated in external datasets, there is still the problem of preference, if the data from multiple databases are intersected, more robust results may be obtained. In addition, although the key genes and their associations with disease progression have been validated based on a large amount of real clinical data from the GEO database, the results would be more convincing if wet experimental validation is added.

An accurate prognostic model based on 10 characteristic pyroptosis-related genes with prognostic values has been constructed for Colorectal adenocarcinoma (COAD). The model can be used to differentiate high-risk samples from low-risk samples, predict metastasis as an independent prognostic factor. And 6 key genes which play key roles in COAD were identified, these genes could potentially be used as drug targets for COAD. Furthermore, the tumor immune microenvironment is a determinant of response to cancer. DCs and neutrophils were infiltrated significantly more in the low-risk group compared with the high-risk group. Reshaping the tumor immune microenvironment by enhancing activations of DCs and neutrophils would be an effective therapy for COAD.

## 5. Conclusion

Pyroptosis is closely associated with cancer. In this study, 10 pyroptosis genes with prognostic values (*CHMP7*, *CHMP6*, *CASP6*, *TP53*, *TNF*, *IRF1*, *PKN1*, *NOD1*, *CHMP4A*, and *GPX4*) were screened based on a constructed genes set. A prognostic model for COAD was constructed based on these genes. The ability to differentiate high-risk samples from low-risk samples, predict survival rate, and predict metastasis of the model was validated using the TCGA and external GEO datasets. The survival curve showed that the survival rate was significantly higher in the low-risk group than it in the high-risk group, the AUCs were obtained as 0.685 based on the survival information of COAD patients at 1 year in the GSE17536 dataset. The sensitivities of the model in predicting metastasis were 61.7% and 80% using the GEO external datasets GSE41258 and GSE40367, respectively. Compared with the abilities of clinically used CEA mRNA, the predictive ability of Riskscore was improved.

The study further analyzed the significant differences in specific pathways and TME between the 2 groups to illustrate the possible mechanisms of prognosis and provide assistance for treatment. In the high-risk group, the study found that transmission across the chemical synapses pathway was associated with a high prognostic risk of COAD for the first time, with *ALDH5A1* as the key gene. Some studies identified the gene *ALDH5A1* as being up-regulated in COAD, but its mechanism was unknown. The study suggested that *ALDH5A1* was involved in the degradation of GABA via transmission across the chemical synapses pathway to produce high concentrations of succinic acid, which was broadly known to be helpful for tumor growth and metastasis. In addition, the study also further confirmed and systematically illustrated the pro-COAD role of the Wnt signaling pathway and its key genes, *DVL3* and *NRCAM*. All the above results were validated by external GEO datasets. In the low-risk group, this study confirmed the suppressive effect of the up-regulated IL-4/IL-13 pathway and its key gene *RORC* in COAD. Based on this finding, we further systematically revealed the reasons for the different outcomes or even the opposite outcomes in the existing research results after the up-regulation of RORC, which is differentially regulated by the TME. To be more specific, the over-expression of *RORC* would polarize primary CD4^+^ T cells into Th17 cells. Th17 cells, regulated by IL-1β, could suppress tumor growth by inducing the expression of chemokines, activating, and recruiting cells such as DCs. However, Th17 cells exerted a pro-COAD effect by secreting interleukins when TGF-β is present, leading to angiogenesis and immunosuppression. Th17 cells may also convert to Th1 cells in an environment with decreased lymphocytes and absent TGF-β. Th1 cells released TNF-α, which then stimulated neutrophil recruitment, finally playing an inhibitory role in tumor growth. Th1 cells could also release IFNγ, which further activated the key gene *PSMB9*. The immunoproteasome composed of the production of *PSMB9* helped present antigens, triggering the recognition and destruction of cancer cells by CD8^+^ T cells. In addition, previous studies had confirmed that the over-expression of *TNFRSF17* could prevent COAD by experiments, however, the specific mechanisms had not been explored. We proposed that the mechanism by which *TNFRSF17* plays a suppressive role in the low-risk group may be through its involvement in the intestinal immune network for the IgA production pathway. Up-regulated expression of *TNFRSF17* promoted the secretion of IgA, which in turn killed COAD cells mediated by cytotoxicity, inhibiting proliferation and metastasis of COAD cells. The above results were also validated by external datasets and immune infiltration analysis.

## Acknowledgments

We thank all the researchers and staff working for The Cancer Genome Atlas database and Gene Expression Omnibus database.

## Author contributions

**Conceptualization:** Mengxi Liu.

**Data curation:** Jin Zhang, Yu Zhao.

**Formal analysis:** Jin Zhang.

**Methodology:** Jin Zhang.

**Project administration:** Mengxi Liu.

**Software:** Jin Zhang.

**Supervision:** Jin Zhang, Xiaoyi Zhang.

**Validation:** Yu Zhao.

**Visualization:** Yu Zhao.

**Writing – original draft:** Mengxi Liu, Xiaoyi Zhang.

## Supplementary Material


